# Retraction: The HBV core protein and core particle both bind to the PPiase Par14 and Par17 to enhance their stabilities and HBV replication

**DOI:** 10.3389/fmicb.2026.1800019

**Published:** 2026-02-06

**Authors:** Frontiers Editorial Office

The journal retracts the article cited above.

Following publication, concerns were raised regarding the integrity of the images in the published figures.

Multiple instances of duplicated and manipulated images have been identified within the manuscript. Figure 1C and 8C appear to be duplicated and flipped 180 degrees, while Figure 9B and E contain duplicated bands. Additionally, Figure 6C of the manuscript appears to be duplicated with Figure 11C from an article titled “PIN1 and PIN4 Inhibition via Parvulin Impeders Juglone, PiB, ATRA, 6,7,4′-THIF, KPT6566, and EGCG t…” Furthermore, a duplication was highlighted between Figure 8C of the manuscript and Figure 6A of an article published in the Journal of Virology.

The authors failed to provide a satisfactory explanation during the investigation, which was conducted in accordance with Frontiers' policies. As a result, the data and conclusions of the article have been deemed unreliable and the article has been retracted.

This retraction was approved by the Chief Editors of Microbiology and the Chief Executive Editor of Frontiers. The authors do not agree with this retraction.

